# Photoelectrochemical Water Splitting Reaction System Based on Metal-Organic Halide Perovskites

**DOI:** 10.3390/ma13010210

**Published:** 2020-01-03

**Authors:** Dohun Kim, Dong-Kyu Lee, Seong Min Kim, Woosung Park, Uk Sim

**Affiliations:** 1 Department of Materials Science and Engineering, Chonnam National University, Gwangju 61186, Korea; ysgb5489@gmail.com (D.K.); dk.lee2324@gmail.com (D.-K.L.); 2 Department of Bioengineering, School of Engineering, The University of Tokyo, 7-3-1 Hongo, Bunkyo-ku, Tokyo 113-8656, Japan; kimsmnice@gmail.com; 3 Division of Mechanical Systems Engineering, Institute of Advanced Materials and Systems, Sookmyung Women’s University, Seoul 04310, Korea

**Keywords:** metal-organic halide perovskite, photoelectrochemical reaction, water splitting, passivation

## Abstract

In the development of hydrogen-based technology, a key challenge is the sustainable production of hydrogen in terms of energy consumption and environmental aspects. However, existing methods mainly rely on fossil fuels due to their cost efficiency, and as such, it is difficult to be completely independent of carbon-based technology. Electrochemical hydrogen production is essential, since it has shown the successful generation of hydrogen gas of high purity. Similarly, the photoelectrochemical (PEC) method is also appealing, as this method exhibits highly active and stable water splitting with the help of solar energy. In this article, we review recent developments in PEC water splitting, particularly those using metal-organic halide perovskite materials. We discuss the exceptional optical and electrical characteristics which often dictate PEC performance. We further extend our discussion to the material limit of perovskite under a hydrogen production environment, i.e., that PEC reactions often degrade the contact between the electrode and the electrolyte. Finally, we introduce recent improvements in the stability of a perovskite-based PEC device.

## 1. Introduction

With continuously increasing energy consumption and pollution levels, enormous efforts have been made through international collaborations in various aspects to tackle energy challenges since the Kyoto Protocol and the Paris Climate Change Accord [1]. One major approach is to minimize the use of carbon-based energy sources, such as gas, coal, and natural gas, since such materials produce greenhouse gas as byproducts. Hydrogen has long been considered the most promising energy source as an alternative to existing carbon-based energy sources due to its high energy density and the cleanness of its energy conversion process. However, existing methods of hydrogen production, including thermochemical production, are often associated with the consumption of huge amounts of fossil fuel or with other forms of environmental damage. Therefore, the eco-friendly production of hydrogen is considered the main challenge in the development of hydrogen-based technology.

Electrochemical water splitting is a potential candidate for the sustainable and environmentally-friendly production of hydrogen fuel [2,3,4,5]. In addition to electrochemical methods, photoelectrochemical (PEC) water splitting has also been drawing attention as an environmentally-friendly hydrogen production method, as it enables very active and stable water splitting using light-sensitive semiconductors and molecular catalysts. Though metal oxides have been extensively researched as PEC water splitting photoanodes due to their long-term stability, a metal oxide having a large bandgap possesses a low photocurrent, as it is unable to sufficiently absorb the wavelengths of the visible light region [6,7,8]. Research has been conducted on the photoelectrochemical water splitting potential of perovskite, as a substitute for metal oxide, due to its potential for bandgap engineering and absorbing various wavelengths. Additionally, perovskite has a high absorption coefficient, long electron and hole diffusion length, and excellent electrical as well as optical properties. Hence, perovskite is gaining attention both as a next-generation solar cell material as well as a PEC cell material [9,10,11,12]. Also, perovskite-based PEC cell has advantages in most processes such as spin coating, and it can be deposited in a short time at low temperatures [13]. Despite outstanding properties, perovskite has a major drawback due to degradation caused by water. When Perovskite comes in contact with water, hydrolysis reactions occur, causing perovskite degradation and decreased performance [14,15]. While PEC requires the electrode and the electrolyte to be in contact with each other, degradation occurs when the perovskite and aqueous solution come into contact. Thus, several researchers have been investigating various passivation technologies for the long-term stability of perovskite-based PEC devices [16,17,18]. In this mini-review, we discuss recent progress on perovskite materials as promising candidates for PEC water splitting reactions and stability issues under aqueous operation, as well as the intrinsic properties about perovskite materials.

## 2. Properties of Materials and Mechanisms of the PEC Cell

### 2.1. Intrinsic Properties of Perovskite Materials

The general molecular formula of perovskite is ABO_3_; perovskite materials have a cubic lattice-nested octahedral layered structure. These materials have superior magnetic, ferroelectric, electrical, and optical properties, attracting attention for potential use in optoelectronic devices. At present, many research groups are actively investigating these characteristic properties, and recently, nuclear magnetic resonance (NMR) spectroscopy has been significantly used for analyzing the structure of these perovskite materials [19]. Perovskite materials also have low recombination probability and high carrier lifetimes and diffusion lengths [20,21]. Therefore, metal-organic halide perovskites have been amongst the most interesting subjects in optoelectronic materials research, and are utilized in various fields such as light-emitting diodes, solar cells, lasers, and photodetectors [22,23,24,25].

**Figure 1:**
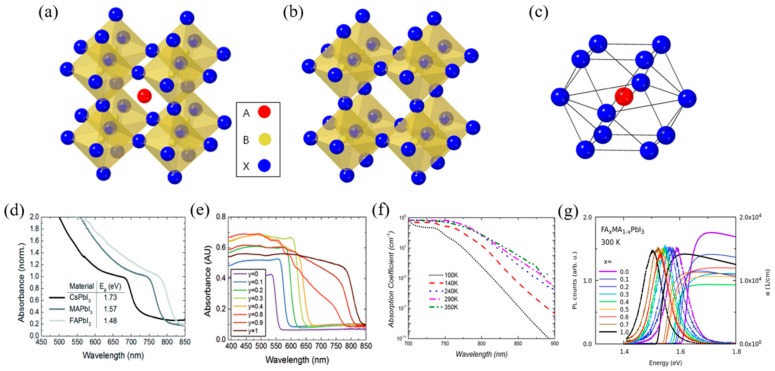
Intrinsic crystal structure and optical properties of perovskite materials. (**a**) A typical perovskite crystal structure. (**b**) Crystal structure of B cation and X anion (B cation is located at the octahedral site of X anion). (**c**) Crystal structure of A cation and X anion (A cation is located at the octahedron site of X anion. (**d**) UV-vis spectra for the APbI_3_ perovskites formed, where A is either cesium (Cs), methylammonium (MA) or formamidinium (FA). (**e**) UV-vis absorption spectra FAPbI_y_Br_3-y_ perovskites. (**f**) Temperature dependence of the absorption coefficient of methylammonium lead iodide (MAPbI_3_) extracted from photoluminescence (PL) spectra. (**g**) Near-bandgap absorption and photoluminescence spectra at room temperature mixed-organic perovskites [39,40,43,44].

The metal-organic halide perovskite has a molecular structure of type ABX_3_, where A and B are cations (A is larger than B), and X is the anion. The general crystal structure of perovskite is shown in Figure 1a–c [26,27]. Perovskites commonly have unit cells consisting of five atoms in a cubic structure (α phase), where the A cation (methylammonium, CH_3_NH_3_^+^, MA^+^, or formamidinium, CH(NH_2_)^2+^, FA^+^) is surrounded by twelve X anions (Cl^−^, Br^−^, or I^−^, or a coexistence of several halogens) to form a cuboctahedron, and the B cation (Pb^2+^, Sn^2+^, etc.) is located at the octahedral site of X. The B cation–X anion octahedra are joined together to form stable three-dimensional network structures [21,28]. To achieve a cubic structure with a high degree of high symmetry, i.e., an ideal perovskite crystal structure, the radius ratio of A, B, and X should be such that tolerance factor {t = (R_A_ + R_B_)/$\sqrt{2}($R_B_ + R_X_)} is close to 1 [29]. For the tolerance factor to approach 1, the A cation must be much larger than the B cation. In metal-organic halide perovskite, the A site must be occupied by a very large atom because the B site is usually occupied with a large atom such as Pb or Sn. Tolerance factors of cubic structures are generally between 0.89 and 1, where a tolerance factor lower than 0.89 could induce a tetragonal (β phase) or orthorhombic (γ phase) structure, whereas higher tolerance factors could induce a two-dimensional (2D) layer structure due to the unstable three-dimensional (3D) B–X bonding [7,30,31]. Actually, the transverse phonon easily displaces the X anion from the B–B intermediate location of the cubic structure [32,33]. The non-perovskite δ phase appears in perovskite materials such as HC(NH_2_)_2_PbI_3_, FAPbI_3_, CsPbI_3_, and CsSnI_3_, which, unlike the β and γ phases, is caused not by B–X–B angle distortion in the α phase, but by the breaking of the B–X bond [34,35,36].

As one of the unique properties of metal-organic halide perovskites, the optical properties of photo-generated charge carriers have been researched. The specific excitonic absorption peaks of the metal-organic halide perovskite could be transited to various absorption spectra, and it changed significantly in visible light through the adjustment of metal atoms and halogens (Figure 1d,e). Converting the metal atom directly changes the M–X bonding, resulting in changes in valence band maximum and conduction band minimum, as the band edge is determined by the metal orbitals of the B site. In fact, in perovskite, the valence band is composed of 5p orbitals of I and the s-antibonding states of Pb 6s. The conduction band is composed of 5s orbital of I and the s-antibonding states of Pb 6p. For example, when the B site is changed from Pb to Sn in MAPbI_3_, the bandgap changes from 1.57 to 1.17 eV, and therefore, the bandgap between 1.57 eV and 1.17 eV can be controlled by adjusting the ratio of Pb and Sn [37,38]. Additionally, converting the A organic cations changes the length and angle of the M–X–M bonding, which, in turn, changes the bandgap but does not affect the valence band maximum. In fact, MAPbI_3_ shows a rapid absorption rise at 825 nm (1.5 eV) and has a large absorption coefficient of 1.8 × 10^4^ cm^−1^. By substituting the methylammonium (MA) cation with formamidinium (FA), the energy bandgap could be lowered to 1.48 eV, which reduces the bandgap by 0.09 eV and allows for additional light absorption [39]. Metal-organic halide perovskites are widely used in the optoelectronics field due to their excellent and wide-ranging absorbance in the visible light range [40]. Furthermore, metal-organic halide perovskites could exhibit amplified spontaneous emission due to the low defect density and slow Auger recombination, even in the presence of electron and hole extinctions [41,42]. The absorption coefficient of perovskite is affected by temperature, tending to decrease at lower temperatures. This is associated with the interaction of excitons and phonons at low temperatures and the phase transition of MAPbI_3_ at about 160 K (Figure 1f) [43]. Similar to light absorption, the photoluminescence (PL) of metal-organic halide perovskite could be tuned by changing organic cations or anions. In the case of MAPbI_3_ quantum dot (QD), the PL spectrum could be tuned from 407 to 734 nm by changing the composition of anions (Figure 1g) [44]. Due to its properties to tune the strong PL spectrum, perovskite is utilized in a variety of light-emitting applications including lasers, light-emitting diodes, and optical sensors [39,45,46,47].

A time-dependent photoluminescence (TDPL) measurement was performed to determine the carrier lifetime (τ) of single crystal FAPbI_3_. As a result, a peak was observed at 820 nm. The two-exponential decay showed fast (τ_1_ = 32 ns) and slow (τ_2_ = 484 ns) carrier lifetimes [15]. A short lifetime represents a high trap density on the crystal surface, while a long lifetime represents carrier transporting in bulk with fewer defects. Generally, FAPbI_3_ has a lower carrier lifetime than single-crystal MAPbI_3_ [48,49,50]. In the case of carrier mobility, it shows change with the perovskite phase transition. The mobility was measured at 35 cm at room temperature; the mobility tends to decrease with increasing temperature at all three steps. Most experimental values follow the T^−3/2^ dependency without discontinuity in phase transitions, and show 150 cm^2^ V^−1^ s^−1^ at 80 K. Mobility is relatively high when the temperature is above room temperature or when entering the cubic phase [10,51,52,53,54]. In addition, the change of carrier mobility is shown by the perovskite layer thickness. Zhang et al. [55] characterized the photovoltaic power conversion efficiency (PCE) with different MAPbI_3_ layer thicknesses to investigate the effect of film thickness on perovskite solar cell devices. The PCE increases as the film thickness approaches 300 nm and tends to decrease rapidly in the 300–530 nm range. The PCE has a maximum of about 10.16% at 300 nm thickness and decreases to 5.29% at 530 nm. This allowed us to determine the optimal thickness of the MAPbI_3_ layer. When thin, pinholes result in low V_OC_ and shunt resistance, while increasing the thickness of the film eliminates this contact, thereby improving V_OC_. J_SC_ is affected by light-harvesting efficiency(η_lh_) and carrier injection efficiency(η_inj_). The increase in J_SC_ with increasing thickness up to 300 nm indicates that the increase in the absorber layer thickness results in higher absorption and higher η_lh_ [56,57,58].

### 2.2. Principle of Photoelectrochemical Water Splitting Reaction

The electrochemical reaction is based on the junction of the electrode and the electrolyte. In electrochemical water splitting, a hydrogen evolution reaction (HER) occurs at the cathode, and an oxygen evolution reaction (OER) occurs at the anode. The oxidation and reduction reactions for water splitting are as follows.

2H_2_O + 2e^−^→H_2_(g) + 2OH^−^, Eo = 0.00 V (HER) (1)

2OH^−^→1/2 O_2_(g) + H_2_O + 2e^−^, E = 1.23 V (OER) (2)

The theoretical potential required for electrochemical water splitting is 1.23 V. However, due to the activation energy required for the reaction, more than 1.23 V is required in practice. In other words, the actual response requires a theoretical value of 1.23 V or higher. The additional potential required is called “overpotential”. Researchers are continuously endeavoring to reduce the overpotential [59,60].

**Figure 2:**
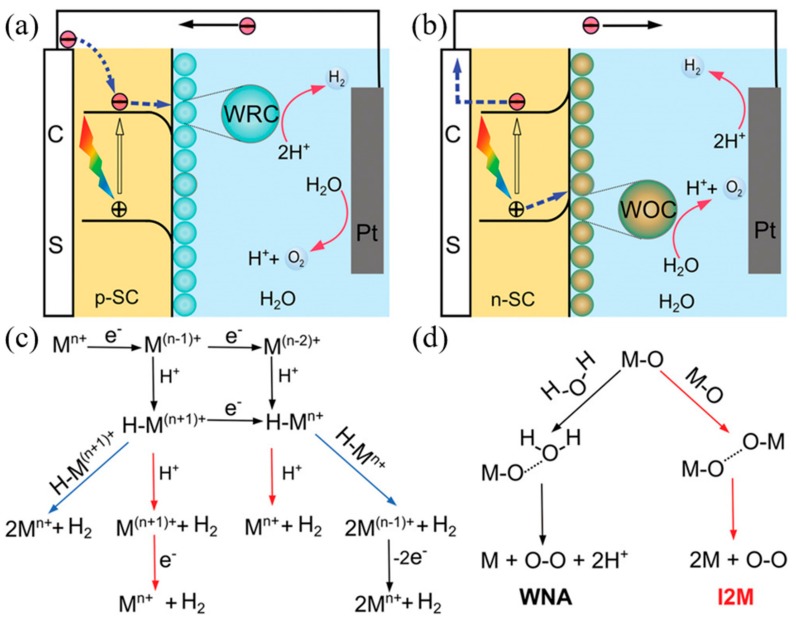
Photoelectrochemical processes involved in (**a**) water reduction and (**b**) water oxidation. (**c**) Proposed mechanistic pathways for H_2_ generation at a metal center of M^n+^. (**d**) Two mechanistic pathways to form an O–O bond for the molecular catalysts. (ref. [61]).

The mechanism by which photoelectrochemical water splitting occurs is shown in Figure 2. Minority carriers generated by semiconductor light absorption are induced into the solution by the electric field at the junction. The electrons and holes generated by the semiconductor are transferred to the molecular catalyst to induce the OER and HER reactions, where, typically, p-type semiconductors are used for water reduction and n-type semiconductors are used for water oxidation. Water reduction is caused by one or two electron steps at the metal center M^n+^, which is followed by the reduction of M^n+^ and, subsequently, followed by protonation, to give an intermediate hydride. Monometallic and bimetallic pathways occur independently or in parallel, and depend on the catalyst properties, its reduction potential, pK_a_ for deprotonation, and pH. In water oxidation, O–O-forming mechanisms are induced by the interaction between two M–O groups (I2 M) or nucleophilic attack by water (WNA). I2 M mechanisms are achieved by reductive coupling and reductive removal, or by radical coupling through inner/intramolecular pathways. In the WNA mechanism, when the M–O is sufficiently electrophilic, the water molecules attack the M–O to form O–O bonding [61,62,63].

### 2.3. Mechanism of Perovskite PEC Cells

The perovskite photoelectrochemical cell consists of a perovskite light absorption layer, an electron transporting layer, and a hole transporting layer (HTL) to extract the generated electron and hole, and a passivation layer to prevent perovskite degradation in aqueous solution. A simplified working principle of the device may be described as follows: when the light falls on the device, the perovskite layer absorbs the light and generates excitons. The electron and hole pairs (EHPs) are created by the thermal energy, which is diffused and separated through the electron and hole transporting layer, respectively [64]. The performance of the perovskite-based device is influenced by the diffusion length, lifetime, and mobility of the generated carriers. The diffusion length of the perovskite depends on the qualities such as the crystallinity and grain size of the perovskite film, indicating that the diffusion length varies based on the perovskite preparation method. In the case of MAPbI_3_, as the diffusion length of the hole is longer than that of the electron, mesoporous TiO_2_ is used to compensate for this short electron diffusion length. Furthermore, performance is improved when the injected charge mobility is fast. Therefore, designing the charge transporting materials and thickness in consideration of the diffusion length and mobility of the charge is one way to improve performance [9,65,66].

The basic function of the electron transporting layer is to enhance the transport of photo-generated electrons through electron-selective contact with the perovskite layer and to prevent hole injection, so as to improve carrier separation and reduce recombination. Generally, TiO_2_, ZnO, [6,6]-phenyl-C_61_-butyric acid methyl ester (PCBM), etc. are used as electron transporting materials. Though TiO_2_ is the most commonly used inorganic electron transporting layer (ETL) material and its electron injection rate is very fast, it has limitations, as recombination occurs due to low electron mobility [67,68,69]. While ZnO has higher electron mobility than TiO_2_ (Bulk mobility: 205–300 cm^2^ V^−1^ s^−1^), its chemical instability is a drawback [70,71]. PCBM is a conductive polymer material with low photocurrent hysteresis and high short circuit current density. PCBM plays a critical role in improving the quality of the light-absorbing layer by filling the pinholes and vacancies between perovskite grains, resulting in a film with large grains and fewer grain boundaries [72,73,74].

The main function of the hole transporting layer is to improve electron-hole pairs separation by collecting and transporting the generated hole from the perovskite layer. The material used for the hole transporting layer is spiro-OMeTAD, poly (3,4-ethylene dioxythiophene) polystyrene sulfonate (PEDOT:PSS), and CuI. Although Spiro-OMeTAD is one of the best performing HTL materials, its cost can be an impediment, and it is also a small molecule substance which diffuses well into perovskite [75,76]. PEDOT:PSS also exhibits good performance and is a conductive polymer material like PCBM. Alternatively, while CuI has about five times the hole mobility of spiro-OMeTAD and a relatively large particle size, signifying that it does not readily diffuse into the perovskite layer, its shortcomings are that the surface is rough and it is not well bonded with perovskite due to the large particles [77,78].

**Figure 3:**
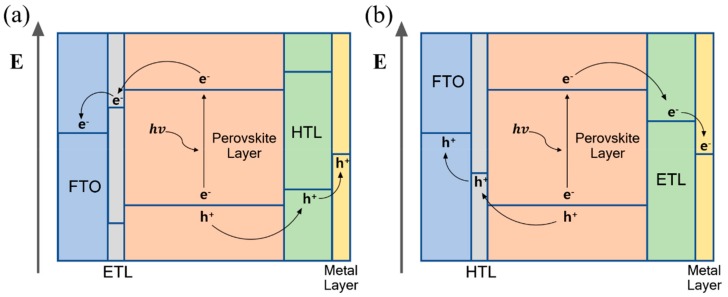
The operating mechanism of perovskite-based photoelectrochemical device (**a**) n–i–p configuration, (**b**) p–i–n configuration.

Perovskite-based device configuration is derived from the dye-sensitized solar cell, and is classified into two types. One of them comprises the transparent conductive substrate coated with the electron transporting material (mesoporous/planar n-type material) under the perovskite layer and hole transporting layer (p-type material) deposited on the perovskite layer. This configuration, which is called n–i–p type, strives for an oxygen evolution reaction. In n–i–p type devices, electron-hole pairs (EHPs) generated in the perovskite layer are transported by the electron transporting material to the transparent conductive oxide (TCO) substrate, and through the hole transporting material, the holes are transported to the metal electrode (Figure 3a). Holes that reach the metal electrode through this mechanism react with oxygen ions to produce oxygen. The other common configuration is inverted compared to the former, and the transparent conductive substrate is coated with the hole transporting material, followed by the perovskite layer and electron transporting layer. This configuration, which is called p–i–n type, strives for a hydrogen evolution reaction. In the p–i–n type perovskite photoelectrochemical cell, a hydrogen evolution reaction occurs by moving electron-hole pairs (EHPs) in a converse mechanism to that of n–i–p type perovskite PEC devices. The EHPs generated in the perovskite layer are transported by the hole transporting material to the TCO substrate, and through the electron transporting material, the electrons are transported to the metal electrode (Figure 3b). The electrons are transported to the metal electrode to produce hydrogen through the reaction with the electrolyte.

## 3. Recent Progress on Perovskite-based Photoelectrochemical Cells

### 3.1. n–i–p Configuration Perovskite-Based PEC Cell for Oxygen Evolution

As described above, n–i–p configuration, perovskite-based PEC cells strive for oxygen evolution by transporting the holes generated in the perovskite layer to the top metal electrode through the hole transporting layer. In the planar type, compact-TiO_2_ is mainly used as an electron transporting layer, which functions as a means of hole blocking to reduce recombination as well as electron transport. In the mesoporous type, mesoporous TiO_2_ or Al_2_O_3_ is deposited onto the compact TiO_2_ to facilitate the formation of the perovskite film.

**Figure 4:**
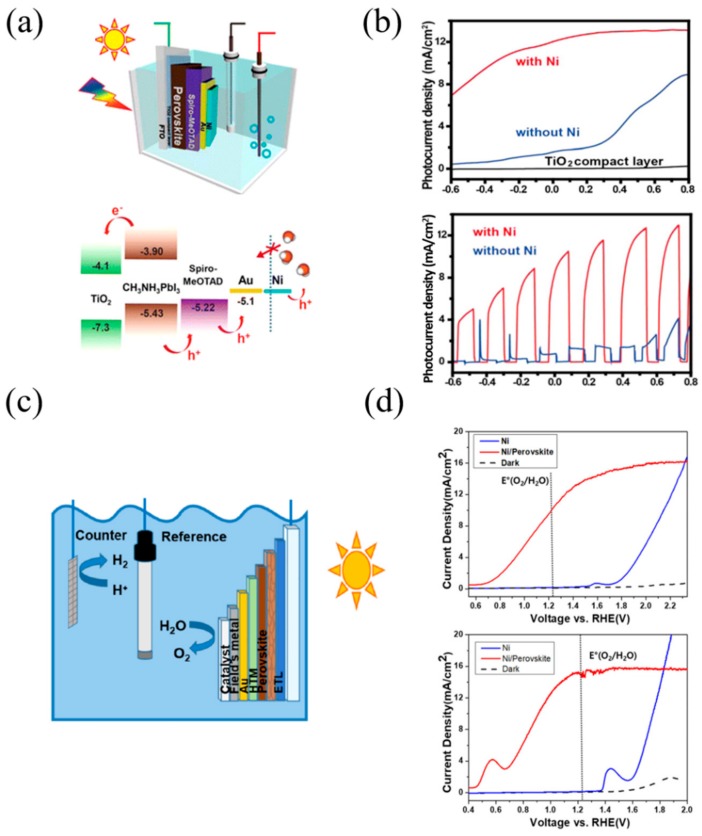
Examples of the n–i–p configuration of perovskite-based PEC cells. (**a**) Schematic illustration of the photoelectrochemical test of a Ni-coated perovskite photoanode in a PEC cell using a standard three-electrode system and energy diagram of each material. (**b**) Photocurrent densities of the compact TiO_2_ layer (black curve) and CH_3_NH_3_PbI_3_ photoanodes without (blue curve) and with Ni surface layer (red curve). Comparison of photocurrent densities of CH_3_NH_3_PbI_3_ photoanodes (with and without Ni surface layer). (**c**) Schematic illustration of the integrated photoelectrolysis cell with perovskite photoelectrode. (**d**) Voltammogram of perovskite photoanode with 10 μm Ni catalyst under simulated illumination (0.7 sun; red curve), under dark (dashed curve), and on metallic Ni electrode (blue curve) as reference. Electrolyte was K-borate solution (pH 9.2) and 1.0 M KOH solution (pH 14) respectively [79,80].

Da et al. [79] designed a perovskite photoanode device with an FTO/TiO_2_/CH_3_NH_3_PbI_3_/Spiro-OMeTAD/Au/Ni configuration (Figure 4a). The perovskite layer was deposited by a one-step method using anti-solvent, and the device consisted of an approximately 50 nm thickness of compact TiO_2_ layer, 220–250 nm thickness of perovskite layer for absorbing light, 250 nm thickness of a spiro-OMeTAD layer, and 80 nm and 8 nm thickness of Au and Ni layer, respectively. The holes generated in the perovskite layer are transferred to the Au/Ni layer through spiro-OMeTAD, and are used for the oxygen evolution reaction. The top Ni layer not only prevents electrolyte from penetrating into the perovskite layer, but also acts as an effective oxidation catalyst. The perovskite photoanode with an ultrathin Ni layer has a high photocurrent density and high stability in alkaline solution. Current-voltage (J-V) tests were performed for the photovoltaic performance, and the device presented the following performance: open-circuit voltages (V_oc_) of ~0.95 V; short-circuit current density (J_sc_) of ~19.0 mA/cm^2^; and a fill factor (FF) of ~0.59. Perovskite-based photoanodes with and without a Ni layer were compared by PEC measurement using the three-electrode system. The PEC measurement confirmed the photocurrent of the photoanode with Ni layer enhancement at overall voltage ranges (the photocurrent density was more than 10 mA/cm^2^ at 0 V vs. Ag/AgCl). This confirms that the Ni layer is not only the passivation layer, but also an efficient catalyst (see Figure 4b).

Nam et al. [80] designed a perovskite photoanode device with a FTO/TiO_2_/CH_3_NH_3_PbI_3_/Spiro-OMeTAD/Au/Field’s metal/Ni configuration (Figure 4c). The device could perform ultrastable solar water splitting, even in a strong alkaline solution, through the deposition of the eutectic Field’s metal (with a eutectic alloy of Bi and Sn, and a low melting point of 62 °C) using a versatile mold-cast and lift-off process. Mold-casting and lift-off processes enable metal encapsulation with precise diameters and thicknesses, the creation of a smooth electrode surface, and ease of additional functional layer deposition. Through the current-voltage (J-V) test, the device presented the following performance: open-circuit voltages (V_oc_) of 0.98 V; a short-circuit current density (J_sc_) of 21 mA/cm^2^; a fill factor (FF) of 0.78; and a power conversion efficiency of 15.6%. Since Field’s metal does not act as a catalyst for oxidation, Ni was deposited as an oxidation catalyst on Field’s metal through electrodeposition. The thin Ni film (~10 μm) also prevents the corrosion of Field’s metals, and acts as a catalyst in strong alkaline solutions. The performance of the photoanode was tested in a three-electrode system. At pHs similar to neutral, photoelectrolysis was performed under acidic or alkaline conditions, because the electrode surface pH gradient and electrode kinetics were slower when under acidic or alkaline solutions. As a result of the fast electrode kinetics and the smaller pH gradient in the alkaline solution, the current rises more rapidly in alkaline than in neutral. Overpotential was measured at 0.915 V and 0.519 V for neutral and alkaline, respectively (Figure 4d). The photocurrent of the perovskite photoelectrode becomes saturated at the positive potential region, which is essentially limited by the incident light intensity (the saturation current of 16 mA/cm^2^ at 0.7 sun).

**Figure 5:**
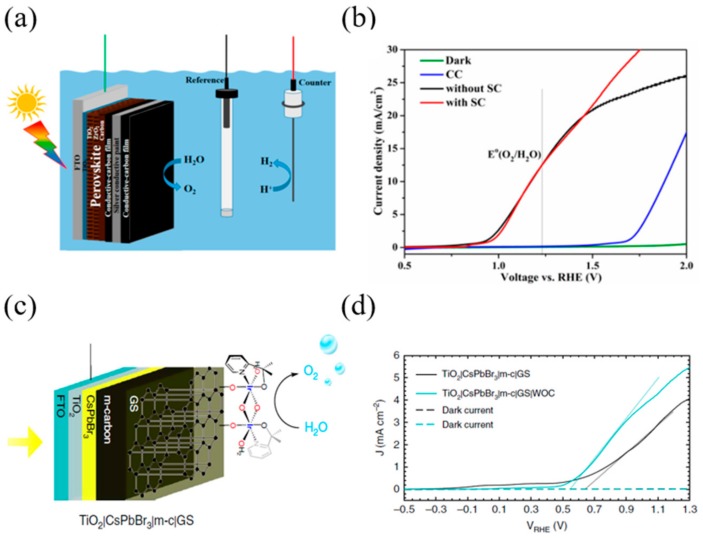
Examples of the n–i–p configuration of perovskite-based PEC cells. (**a**) Schematic illustration of the perovskite photoanode for PEC water splitting in a standard three-electrode system. (**b**) Voltammogram of perovskite photoanodes with SC paint layer (red curve) and without SC paint layer (black curve) under simulated illumination and in the dark (green curve), with the CC paste electrode (blue curve) as a reference. (**c**) Schematic illustration of perovskite photoanode device used CsPbBr_3_, TiO_2_ (electron collecting), m-carbon (hole collecting). (**d**) Linear sweep voltammetry (LSV) of a TiO_2_|CsPbBr_3_|m-c|GS and a TiO_2_|CsPbBr_3_|m-c|GS|WOC measured at a scan rate of 20 mV s^−1^, in a 0.1 M KNO_3_ electrolyte solution with pH 2.5 (pH adjusted with H_2_SO_4_) [81,82].

Tao et al. [81] designed a perovskite photoanode device using a commercial conductive carbon paste (CC) and conductive silver paste (SC) as a hole transporting layer and passivation layer, respectively. Perovskite was used as a mixed-cation perovskite (5-AVA)_x_(MA)_1−x_PbI_3_ (Figure 5a). It was observed that 5-aminovaleric acid (5-AVA) cation facilitates perovskite crystal formation in the mesoporous oxide host, resulting in better surface contact with TiO_2_ and low defect concentrations. Conductive carbon paste (CC) and conductive silver (SC) possess waterproof and hole transporting characteristics. The CC paste layer has a bifunctional characteristic that plays a role in hole extraction and in collection, as its work function is similar to gold. The SC paste layer creates a conductive interface with low resistance between the two CC paste layers, which allows the holes to move more freely. The photogenerated holes from the perovskite layer arrive at the top CC layer through the CC layer and the SC layer, and react with the electrolyte to produce an oxygen evolution reaction. The junction of the two conductive layers with different morphologies protects the underlying perovskite layer entirely, ensuring that the perovskite photoanode has superior performance and stability. A perovskite photoanode PEC test was performed using a three-electrode system; the photocurrent density was approximately 12.4 mA/cm^2^ at 1.23 V (vs. RHE). In order to examine the effect of the SC layer, the test was performed with and without the SC layer (see Figure 5b). Oxygen production through the PEC test was measured by gas chromatography, and the faradaic efficiency of the oxygen evolution of the Photoanode was calculated to be over 80%. This indicates that the increase in photocurrent is the result of oxygen production. The applied bias photon-to-current efficiency (ABPE) through the J-V curve with and without SC was shown to be up to 0.85 and 0.87, respectively. Photoanode devices using conductive carbon pastes do not require complex catalyst deposition processes, and offer superior stability using cheap carbon paste materials.

Poli et al. [82] designed a photoanode that could operate at a wide pH range (2–13) using CsPbBr_3_ perovskite material and a commercial thermal graphite sheet (Figure 5c). The combined layer of 20 µm thick mesoporous carbon (m-c) and commercial hydrophobic graphite sheet (GS) prevented the perovskite layer from being degraded by water. The device could operate in the 2–13 pH range, and the photocurrent at 1.23 V_RHE_ showed close to 3.8 mA/cm^2^ in acidic solution. In addition, the device showed more than 2 mA/cm^2^ current for 30 h and excellent stability in an alkaline electrolyte solution (pH 12.5). By depositing the Ir-based water oxidation catalyst (WOC) on the GS surface, the onset potential of the photoanode was reduced to 100 mV. With and without WOC, samples show similar open-circuit potential and ΔV_ph_ at equilibrium, and similar water oxidation photocurrent at 1.23 V_RHE_ (see Figure 5d). This indicates that the presence of WOC increases the charge transfer kinetics rather than the thermodynamic effect. Finally, the faradaic efficiency for the photoelectrochemical oxygen evolution of a TiO_2_|CsPbBr_3_|m-c|GS|WOC photoanode device was calculated to be 82.3%.

### 3.2. p–i–n Configuration Perovskite-Based PEC Cell for Hydrogen Evolution

As described above, the p–i–n configuration, perovskite-based PEC cell strives for hydrogen evolution by the opposite mechanism of the n–i–p configuration, transporting electrons generated in the perovskite layer to the top metal electrode through the electron transporting layer. In the p–i–n configuration, the electron transporting material cannot use materials requiring high heat treatment such as TiO_2_, and therefore, primarily uses organic materials such as PCBM.

**Figure 6:**
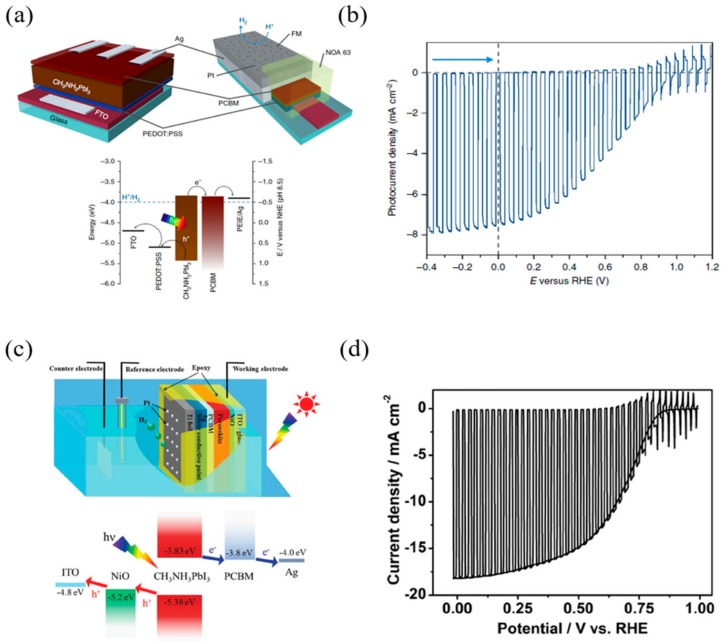
Examples of the p–i–n configuration of perovskite-based PEC cells. (**a**) Material and electronic configuration of the perovskite-based photocathode. (**b**) Linear sweep voltammetry of the perovskite-based photocathode at a scan rate of 5 mV s^−1^. (**c**) Schematic illustration of the sandwich-like CH_3_NH_3_PbI_3_ photocathode for PEC H_2_ evolution in a standard three-electrode system. (**d**) Current density–potential curves for the Pt–Ti/CH_3_NH_3_PbI_3_ photocathode [83,84].

Crespo-Quesada et al. [83] devised a stable perovskite-based photocathode using Field’s metal (a fusible In–Bi–Sn alloy) for the passivation layer (Figure 6a). The perovskite photocathode device had a thickness of 40 nm of PEDOT: PSS, a 300 nm thickness of MAPbI_3_, a 40 nm thickness of PCBM, and a 100 nm thickness of Ag. Field’s metal encapsulation was performed for two purposes: to prevent perovskite degradation from water, and to effectively transport the photogenerated electron. Perovskite-based photocathode photoelectrochemical tests showed that the average photocurrent was −6.9 ± 1.8 mA/cm^2^ at 0 V (vs. RHE), and the average onset potential was 0.95 ± 0.03 V_RHE_ (Figure 6b). Finally, the faradaic efficiency for the hydrogen evolution of the photocathode was calculated to be 95.1 ± 2.2%.

Zhang et al. [84] proposed a perovskite-based photocathode using titanium foil for the p–i–n type structure. The device used NiO as a hole transporting material, PCBM as an electron transporting material, and Ti foil as a passivation layer (see Figure 6c). Titanium is inexpensive, robust, and chemically stable. However, as titanium is difficult to use as an HER catalyst, additional Pt sputtering was performed on the Ti foil. The results of the photovoltaic performance test of the device exhibited a short-circuit current density (J_sc_) of −20.38 mA/cm^2^, an open-circuit voltage (V_oc_) of 1.09 V, a fill factor (FF) of 72.9%, and a power conversion efficiency (PCE) of 16.1%, and the external quantum efficiency (EQE, 390–720 nm range) was calculated to be 70%. The PEC performance measurement of the device indicated a photocurrent of −18 mA/cm^2^ at 0 V_RHE_ and an onset potential of 0.95 V_RHE_, and the ideal ratiometric power-saved efficiency was calculated to be 7.63% (see Figure 6d). The onset potential and photocurrent values of the photocathode through the PEC test were similar to those of V_oc_ and J_sc_, demonstrating that the benefits of perovskite PV devices can be obtained in PEC devices. Finally, the faradaic efficiency for hydrogen evolution of perovskite-based photocathode devices was calculated to be close to 100%.

**Figure 7:**
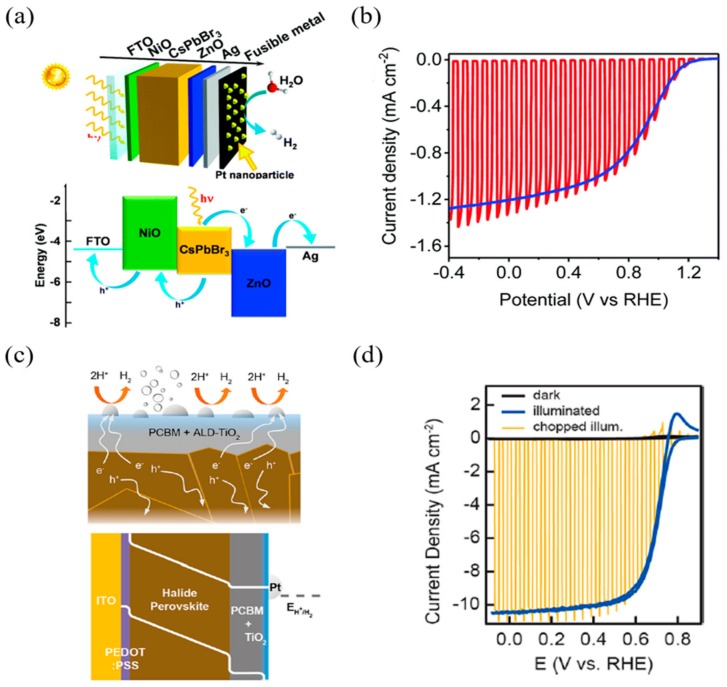
Examples of the p–i–n configuration of perovskite-based PEC cells. (**a**) Schematic representation of a CsPbBr_3_-based photocathode. (**b**) Performance of all-inorganic LHP photocathode under AM 1.5 G simulated sunlight conditions. Current density–potential curves. (**c**) Cross-sectional schematic of a photocathode stabilized against corrosion in 0.5 M H_2_SO_4_(aq) via a hybrid ETL comprising PC_61_BM with TiO_2_ deposited by ALD. Photogenerated electrons are conducted through the TiO_2_ to the Pt catalysts, where electrons are used to reduce protons to hydrogen. (**d**) Photoelectrochemical behavior of the photocathode in the dark and under continuous and chopped illumination reveals the time scale (ms) and magnitude (>10 mA/cm^2^) of the photocurrent response [85,86].

Gao et al. [85] designed a perovskite photocathode using bulk material-based encapsulation (see Figure 7a). To thoroughly prevent the penetration of water into the perovskite layer, the device was encapsulated using a fusible In–Bi–Sn alloy, and silver was deposited between the passivation layer and the electron transporting layer (ZnO) to improve the electrical conductivity. Finally, Pt was deposited on fusible alloys as a catalyst for hydrogen evolution reactions. The results of the photovoltaic performance test of the device exhibited a short-circuit current density (J_sc_) of −2.50 mA/cm^2^, an open-circuit voltage (V_oc_) of 1.37 V, a fill factor (FF) of 55.8%, and a power conversion efficiency (PCE) of 1.91% (Figure 7b). The PEC performance measurement of the device exhibited a photocurrent of 1.2 mA/cm^2^ at 0 V_RHE_ under AM 1.5 G simulated sunlight conditions (100 mW/cm^2^), an onset potential of 1.16 V_RHE_, and a maximum half-cell solar to hydrogen efficiency (HC-STH) of 0.64%. Finally, the faradaic efficiency of the hydrogen evolution of the CsPbBr_3_-based photocathode was calculated to be over 90%.

Kim et al. [86] designed a photocathode using a triple-cation formulation of halide perovskite and a hybrid electron transporting layer (see Figure 7c). Triple-cation perovskite exhibits improved thermal stability and exceptional optical and electrical performance compared to simple methylammonium lead iodide, which, for its part, is expected to improve the overall photoelectrochemical performance; additionally supplemental Cesium (Cs) assists in uniform perovskite film formation. The organic component of ETL, PC_61_BM, shows exceptional electron-transporting and low recombination compared to TiO_2_. Moreover, device stability was secured by depositing TiO_2_ through atomic layer deposition. PEC performance measurement of the device exhibited a photocurrent of 10.5 mA/cm^2^ at 0 V_RHE_ under AM 0.5 G simulated sunlight conditions, the onset potential was more positive than that observed for dark HER under the same conditions, and the fill factor was calculated to be 68.87% (see Figure 7d). This large FF appears to be the result of the fast charge transporting and recombination inhibition of hybrid ETLs. The above cases are summarized in the Table 1.

## 4. Stability Issues of Perovskite PEC Cells

### 4.1. Degradation of Perovskite Material by Moisture

**Figure 8:**
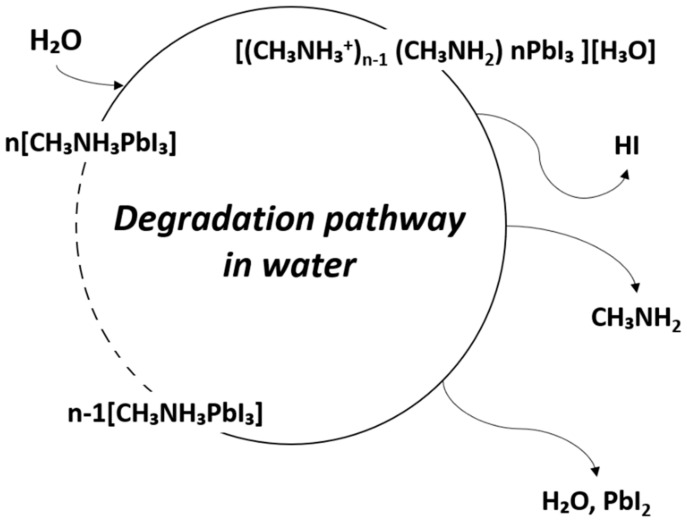
Degradation pathway of metal-organic halide perovskite materials in water.

The instability of metal-organic halide perovskite in humid environments is the biggest obstacle to its applicability in devices, given its other notable properties. The perovskite film is highly sensitive to the presence of water, which affects the stability of devices in which it is used. In addition, perovskite can degrade at the polar solvent. The degradation of perovskite has been reported due to the highly hygroscopic property of amine salts [89]. Due to the high hygroscopicity of organic compounds, when perovskite is exposed to water, organic compounds detach from the crystal structure, and the perovskite crystallinity is changed. This results in changes in the optical and electrical properties causing variations in the performance of perovskite-based devices [90]. In other words, due to the degradation of perovskite by water, the noteworthy optical and electrical properties of perovskite are lost, and the device performance is degraded. The degradation mechanism due to moisture is shown in Figure 8 [91].

CH₃NH₃PbI₃ (s) → CH₃NH₃PbI (aq) + PbI_2_ (s) (3)

CH₃NH₃PbI (aq) → CH₃NH_2_ (aq) + HI (aq) (4)

4HI (aq) + O_2_ (g) → 2I_2_ (s) + 2H_2_O (l) (5)

2HI (aq) → H_2_ (g) + I_2_ (s) (6)

In fact, perovskite readily transforms to monohydrate phase MAPbI_3_∙H_2_O in moderate humidity (RH < 60%), and to dihydrate phase (MA)_4_PbI_6_∙2H_2_O in high humidity (RH > 80%). This can be explained by the fact that the hydrogen bonding interaction between the lead iodide framework and organic MA^+^ cations is weakened by hydration. As a result, MA is diffused and detached from PbI6 octahedra, whereby MAPbI_3_ degrades rapidly. The activation barrier for vacancy-mediated MA^+^ migration is reduced from 1.18 eV for MAPbI_3_ to 0.38 eV for water-intercalation, and 1.14 eV for the monohydrated phase. When MAPbI_3_ is exposed to an aqueous solution, it degrades to PbI_2_ precipitate, iodide anion, and methylammonium cation [92,93].

**Figure 9:**
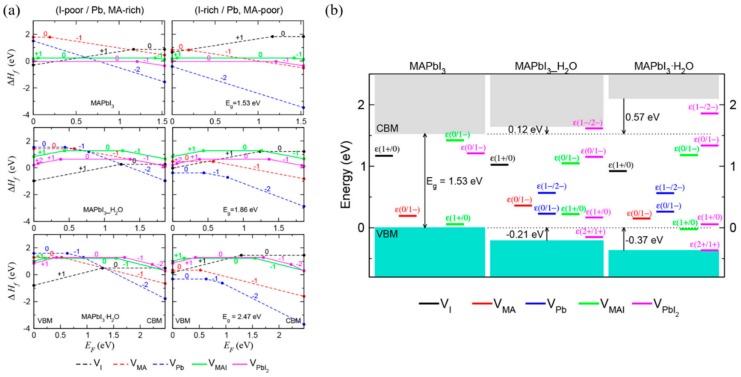
DFT calculation of perovskite’s degradation pathway in water. (**a**) Formation enthalpies of vacancy point and pair defects as a function of the Fermi energy (E_F_) under I-poor (Pb-, MA-rich) conditions (left) and I-rich (Pb-, MA-poor) conditions (right). (**b**) Band alignment and thermodynamic transition levels in MAPbI_3_, water-intercalated MAPbI_3__H_2_O, and monohydrate MAPbI_3_·H_2_O, where deep-lying Pb 5d levels are used as a reference for the VBM and CBM of each phase [92].

Kye et al. [92] identified perovskite degradation in water through a point defect process by density-functional theory (DFT) calculation. Due to the kinetic barrier for I^−^, ion migration becomes very low when hydrated, and V_PbI2_ formation occurs spontaneously. In the hydrous compound, the formation of V_I_ and V_MA_ is preferred to the formation of V_MAI_, so that during the MAPbI_3_ degradation, the formation of I_2_ or CH_3_NH_2_ or HI is higher than that of MAI (Figure 9a). Unlike bulk MAPbI_3_, all vacancy defects form deep transition levels through electrostatic interaction with water molecules (Figure 9b).

Several methods have been identified to reduce the degradation of perovskite solar cells (PSC) by water. The first is to add a thin blocking layer like Al_2_O_3_ between the perovskite and hole transporting material (HTM) [94,95]. The second is to use moisture blocking HTM. The last method is to use hydrophobic carbon electrode [96,97].

### 4.2. Passivation Strategies for Improving Stability

Perovskite-based photoelectrochemical devices require a direct junction between the electrolyte and the device. However, due to the tendency of the perovskite material to degrade when in contact water, which is a vulnerability, passivation technology to protect it is essential. There are two fundamental methods of passivation: (i) bulk material-based encapsulation, and (ii) compact material-based passivation. Passivation using bulk materials prevents moisture from entering the device, primarily by using titanium foil or Field’s metal with a low melting point.

**Figure 10:**
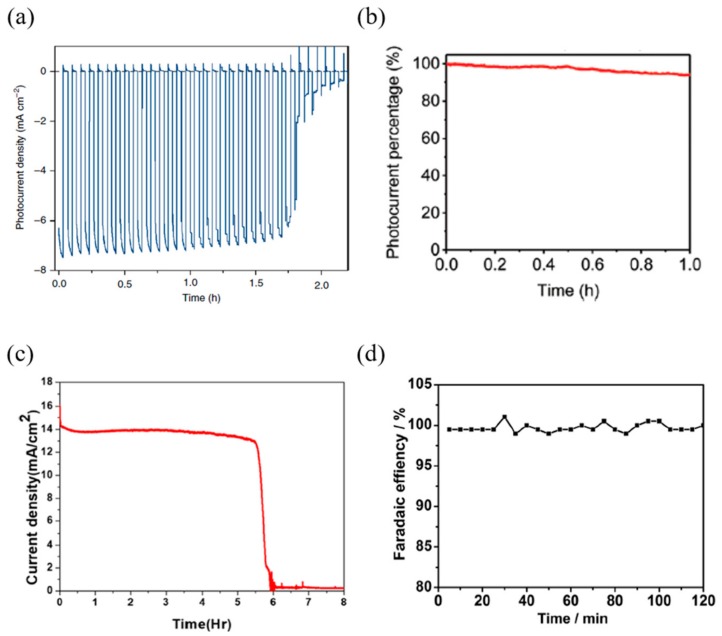
Cases of improving stability by passivation. (**a**) Chronoamperometric trace recorded at an applied potential of 0 V vs. RHE. An aqueous buffer solution (0.1 M borate, pH 8.5), chopped solar light irradiation (AM 1.5 G, 100 mW/cm^2^, λ > 400 nm). (**b**) Stability analysis at 0 V_RHE_. (**c**) Current vs. time graph of perovskite photoanode under illumination (0.7 sun) and bias (1.3 V vs. RHE) in KOH solution (pH 14). (**d**) Faradaic efficiency for hydrogen evolution on the Pt–Ti/CH_3_NH_3_PbI_3_ photocathode with Pt and SCE as the counter and reference electrodes, respectively, in 0.5 M H_2_SO_4_ solution [80,83,84,85].

Crespo-Quesada et al. [83] devised a perovskite-based photocathode employing Field’s metal, which showed a performance decrease of 80% of the initial photocurrent after 1.5 h, and then showed a sharp photocurrent reduction in 0.1 M borate (see Figure 10a). Gao et al. [85] also devised a photocathode for hydrogen production with Field’s metal passivation; the device maintained a stable 94% photocurrent after 1 h under AM 1.5 G simulated sunlight conditions (see Figure 10b). Nam et al. [80] devised a perovskite-based photoanode for oxygen production with the Field’s metal passivation; the device showed superior stability while maintaining an initial photocurrent of 93% after 5 h under AM 0.7 G sun and KOH solution (see Figure 10c). Zhang et al. [84] designed a photocathode device with titanium foil passivation, which showed stable characteristics for 12 h, and notably, exhibited the same hydrogen evolution Faradaic efficiency for 2 h in a 0.5 M H_2_SO_4_ solution (see Figure 10d).

**Figure 11:**
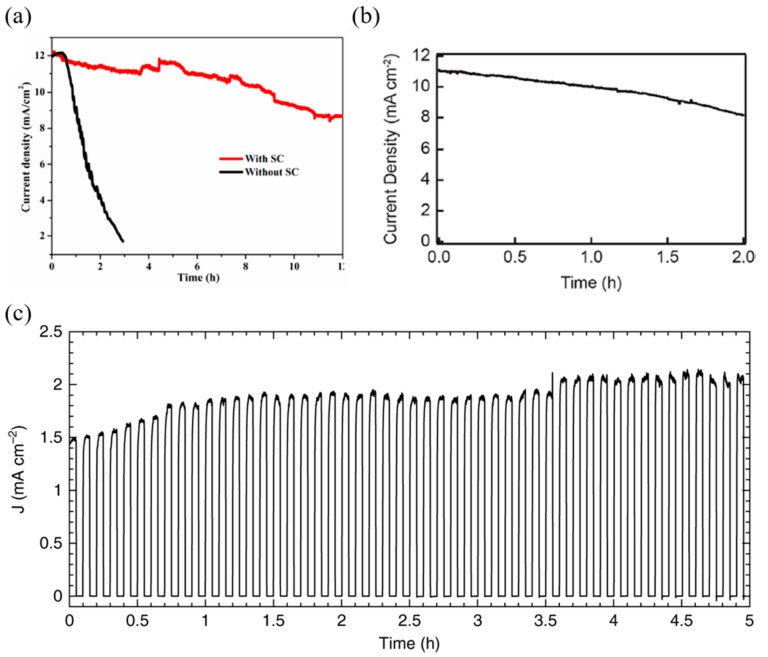
Cases of improving stability by passivation. (**a**) Current versus time graph of a perovskite photoanode at an applied potential of 1.23 V versus RHE under simulated AM 1.5 G solar illumination (100 mW/cm^2^) in 1 M KOH solution. (**b**) Photocurrent density versus time of a photocathode with a nominally 15 nm thick Pt catalyst under continuous illumination. The electrode potential was held at 0 V vs. RHE during continuous illumination of 0.5 Sun. (**c**) Chronoamperometric trace recorded at an applied potential of 1.23 V_RHE_ in KOH electrolyte solution at pH 12.5, under chopped simulated solar light irradiation (AM 1.5 G, 100 mW/cm^2^) [81,82,86].

The second method, i.e., the passivation method using compact materials, prevents water from entering the perovskite layer by means of thin surface treatment. Although Da et al. [79] improved the stability of the photoanode by sputtering ultrathin Ni layers (8 nm), the device showed 56% photocurrent after 100 s through a PEC test in alkaline solution, showing a performance degradation in a short period time. Tao et al. [81] designed a photoanode with both high stability and high performance using conductive carbon paste and silver paste. Carbon paste was deposited directly onto the perovskite layer to enable hole transporting and passivation. However, due to the low stability of the single carbon paste, a combination of carbon paste and silver paste was used to enhance stability, which, in turn, made it possible to maintain more than 70% photocurrent after 12 h of operation (see Figure 11a). Kim et al. [86] improved the stability of the photocathode device by forming a thin, compact layer through titanium atomic layer deposition (ALD); the device showed 75% photocurrent (10.5 mA/cm^2^) in a strong acid solution (H_2_SO_4_), even after 2 h (see Figure 11b). Finally, Poli et al. [82] created a highly-stable photoanode through passivation using a mesoporous carbon and graphite sheet; the device was stable for 5 h in a pH 12.5 solution, and the photocurrent was increased from 1.5 mA/cm^2^ to 2 mA/cm^2^. The device also showed a lifetime of 34 h in alkaline electrolyte, 23 h in a near-neutral solution, and 7.8 h in an acidic solution (see Figure 11c).

### 4.3. Enhanced Stability through the Introduction of Inorganic Perovskites

So far, as the main topic, metal-organic halides have been discussed as perovskite materials composed of organic materials and halides such as bromine and iodine. As mentioned above, they have some key advantages in the manufacturing process of the perovskite layer, because they can be produced at low temperatures, though it is well-known that metal-organic halides are vulnerable to damage from moisture. In contrast, inorganic perovskite materials require high temperatures for synthesis that cause some difficulties in the manufacturing process, but they are more sustainable in the atmosphere, comparatively. Therefore, some researchers are mainly focusing on inorganic perovskite-based PEC systems.

**Figure 12:**
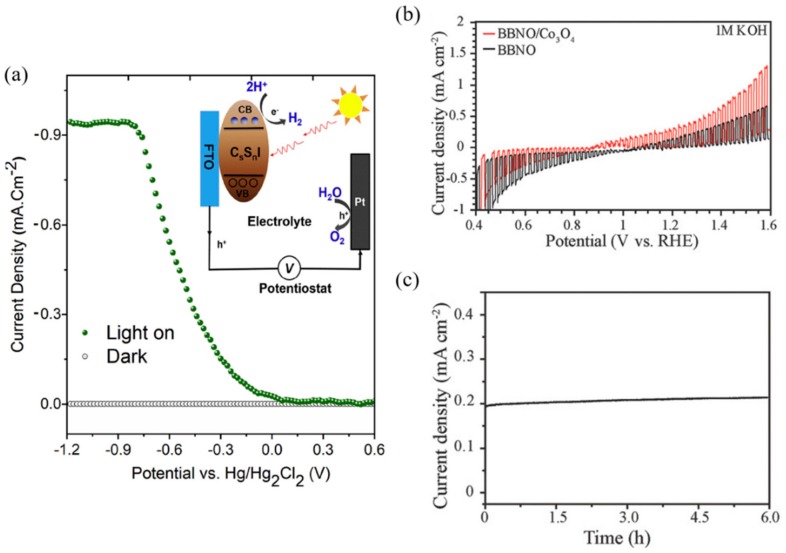
Cases of improving stability by using inorganic perovskites. (**a**) The photocurrent density curve of the Cs_2_SnI_6_ compound using a three-electrode system with a 0.3 M NaCl electrolyte and the inserted picture shows the working principle of the system. (**b**) PEC water splitting performance of a Ba_2_Bi_1.4_Nb_0.6_O_6_ photoanode. LSV curves of PEC water oxidation in 1 m KOH solution of Ba_2_Bi_1.4_Nb_0.6_O_6_ film and Co_3_O_4_ coated Ba_2_Bi_1.4_Nb_0.6_O_6_ film (Pt, as counter electrode and cathode). (**c**) Durability test of the Ba_2_Bi_1.4_Nb_0.6_O_6_ film at 1.23 V (vs. RHE) [98,99].

Dang et al. [98] synthesized an inorganic perovskite Cs_2_SnI_6_ film through a solution process. Cesium tin iodide generally has an orthorhombic structure at room temperature, and this material demonstrates phase transitions under synthesis conditions (ambient air, annealing temperature, synthesis method). The phase transition of the cubic structure has high active site density and a slow rate of intraband relaxation of the excitonic state, which is expected to improve the performance of the PEC system. Here, Cs_2_SnI_6_ was synthesized with a uniform morphology of cubic structures through annealing at 170 °C for 8 h. PEC cells were prepared by synthesizing Cs_2_SnI_6_ on an FTO substrate using a solution-processed approach. The PEC test was conducted in 0.3 M NaCl, and showed a photocurrent density of 0.92 mA/cm^2^ with an energy conversion efficiency of 0.54% at −0.8 V (Hg/Hg_2_Cl_2_) (Figure 12a).

Weng et al. [99] proposed an improved PEC performance by reducing the bandgap through the bandgap engineering of Barium Bismuth Niobate Double perovskite (BBNO) to enhance the absorption of visible light. The results suggest that Bi-rich, Nb-poor off-stoichiometry could reduce the bandgap using density-functional theory (DFT) calculations. In BBNO crystals, the valance band maximum (VBM) is determined by the Bi 6s lone pair orbital (Bi^3+^), and the conduction band minimum (CBM) is determined by the unoccupied Bi 6s orbital (Bi^5+^). In addition, strong antibonding of Bi 6s and O 2p orbital shifts the VBM to higher energy states. On the other hand, because the unoccupied Bi 6s orbital is located at the much lower energy level than the unoccupied Nb 4d and Bi 6p orbitals, the bandgap gets reduced when both Bi^3+^ and Bi^5+^ cations coexist. Ba_2_Bi_1.4_Nb_0.6_O_6_ was confirmed to have the lowest VBM by DFT calculation. Therefore, in the Ba_2_Bi_1.4_Nb_0.6_O_6_ system, the hole would be easily transported to the electrolyte; therefore, a PEC oxidation test was conducted. Thus, the Ba_2_Bi_1.4_Nb_0.6_O_6_ photoanode exhibited 0.2 mA/cm^2^ at 1.23 V_RHE_ and 0.4 mA/cm^2^ photocurrent density at 1.23 V_RHE_ when the Co_3_O_4_ nanoparticle catalyst was deposited (see Figure 12b). Also, it showed a constant photocurrent density for 6 h and excellent stability without any changes in the x-ray diffraction (XRD) patterns (Figure 12c).

In addition, Bin et al. [100] designed a PEC cell with a hybrid tandem structure using an organic photovoltaic, perovskite solar cell, and graphene. The bottom cell consists of polymer with a wide bandgap, while the top cell possesses a MAPBI_3_ perovskite-based cell separated by a layer of graphene. Therefore, the hybrid tandem perovskite solar cell showed a short-circuit voltage (J_sc_) of 8.73 mA/cm^2^, an open-circuit voltage (V_oc_) 1.86 V, a fill factor (FF) 0.72, and a power conversion efficiency (PCE) of 11.28%. The PEC test showed a photocurrent of ~7.25 mA/cm^2^ and 9.02% solar-to-hydrogen efficiency.

## 5. Conclusions

In summary, this review discusses the advancements of perovskite-based photoelectrochemical cells for generating renewable energy. As a result of their exceptional electrical and optical properties, the application of perovskite materials to photoelectrochemical reactions aims to enhance performance. However, it is essential to improve the stability of perovskite by passivation due to the requirement of an aqueous solution. Therefore, research into the development of stable, perovskite-based photoelectrochemical cells which overcome vulnerability to aqueous solutions has been pursued. In order to minimize degradation by encapsulating the device using bulk material, Crespo-Quesada et al. [83], Gao et al. [85], and Nam et al. [80] devised cells using Field’s metal as a passivation layer, and Zhang et al. [84] devised a cell using titanium foil as a passivation layer. Conversely, as a method of using compact material deposition, Da et al. [79] formed a passivation layer by sputtering ultrathin Ni layers (8 nm), and Tao et al. [81] devised a cell using a passivation layer by depositing conductive carbon and silver paste through the doctor blade method. Kim et al. [86] formed a passivation layer through the ALD process, and Poli et al. [82] devised a stable perovskite-based PEC device using mesoporous carbon and a graphite sheet. Nevertheless, the use of perovskite as a PEC requires improved stability through the development of passivation technology, because of its low stability when compared to other PEC materials. Therefore, for the advancement of perovskite-based PEC devices, continuous research into passivation technology for stable PEC reactions, as well as research into performance improvement, is essential. Perovskite-based PEC devices with long-term stability and exceptional optical and electrical properties are expected to enable effective and environment-friendly chemical energy production through PEC reactions.

## Figures and Tables

**Table 1:** Photoelectrochemical and photovoltaic performance of perovskite-based cells depending on charge transporting materials, perovskite materials, and cell configurations. (* Approximate value).

Device Configuration		Passivation Method	Jsc	Voc	FF	PCE	Over Potential	Onset Potential	Stability	Photocurrent	Faradaic Efficiency	Ref
n–i–p type	FTO/TiO_2_/MAPbI_3_/Spiro-OMeTAD/Au/Ni	Ni sputtering	19 mA/cm^2^	0.95 V	0.59	10%	−0.45 V_Ag/AgCl_ *	N/A	100 s—50%	10 mA/cm^2^ (at 0 V_Ag/AgCl_)	N/A	[79]
	FTO/TiO_2_/MAPbI_3_/Spiro-OMeTAD/Au/Field’s metal/Ni	Field’s metal encapsulation	21 mA/cm^2^	0.98 V	0.78	15.60%	−0.489 V_RHE_	0.75 V_RHE_ *	5.5 h—50%	14 mA/cm^2.^(at 1.23 V_RHE_)	N/A	[80]
	FTO/TiO_2_/(5-AVA)_x_(MA)_1−x_PbI_3_/CC/SC/CC	CC/SC paste doctor blade	N/A	N/A	N/A	N/A	0.03 V_RHE_ *	0.8 V_RHE_ *	12 h—70%	12.4 mA/cm^2^ (at 1.23 V_RHE_)	82%	[81]
	FTO/TiO_2_/CsPbBr_3_/m-carbon/GS/WOC	m-carbon/GS doctor blade	5.05 mA/cm^2^	1.4 V	0.749	5.30%	N/A	0.6 V_RHE_ *	30 h—50%	2.5 mA/cm^2^ (at 1.23 V_RHE_)	N/A	[82]
	FTO/TiO_2_/MAPbI_3_/spiro-OMeTAD/Ni-Au/CNT-polymer composite	CNT-polymer composite	18.3 mA/cm^2^	1.06 V	0.74	14.4%	0.85 V_RHE_ *	0.3 V_RHE_ *	10 h *	9.2 mA/cm^2^ (at 1.23 V_RHE_)	N/A	[87]
p–i–n type	FTO/PEDOT:PSS/MAPbI_3_/PCBM/Ag/Field’s metal/Pt	Field’s metal encapsulation	15 mA/cm^2^	1 V	0.54	8%	−0.2 V_RHE_ *	0.7 V_RHE_ *	1.8 h—50%	−9.8 mA/cm^2^ (at 0 V_RHE_)	95.10%	[31]
	ITO/NiO/MAPbI_3_/PCBM/Ag/Silver paint/Ti foil/Pt	Ti foil deposition	20.38 mA/cm^2^	1.09 V	0.729	16.10%	0.69 V_RHE_ *	0.95 V_RHE_	12 h	−18 mA/cm^2^ (at 0 V_RHE_)	100%*	[84]
	FTO/NiO/CsPbBr_3_/ZnO/Ag/Field’s metal/Pt	Field’s metal encapsulation	2.5 mA/cm^2^	1.37 V	0.558	1.91%	N/A	1.16 V_RHE_	1 h—94%	−1.2 mA/cm^2^ (at 0 V_RHE_)	90%	[85]
	ITO/PEDOT:PSS/Cs_0.05_(MA_0.18_FA_0.83_) _0.95_Pb(I_0.83_Br_0.17_)_3_/PCBM/TiO_2_/Pt	TiO_2_ Atomic Layer Deposition	21.4 mA/cm^2^	0.73 V	0.655	9.63%	−0.05 V_RHE_	0.68 V_RHE_	2 h—75%	−10.5 mA/cm^2^ (at 0 V_RHE_)	N/A	[86]
	ITO/NiOx/CsFAMAPbI_3_/PCBM/AZO/FM/Pt	Field’s metal encapsulation	23.15 mA/cm^2^	0.97 V	0.814	18.20%	0.3 V_RHE_ *	0.7 V_RHE_ *	18 h *	−14.3 mA/cm^2^ (at 0 V_RHE_)	85% *	[88]
